# Functional Biomaterials for Local Control of Orthodontic Tooth Movement

**DOI:** 10.3390/jfb14060294

**Published:** 2023-05-25

**Authors:** Yi Lin, Moyu Lara Fu, Ingrid Harb, Lisa Xiaolu Ma, Simon D. Tran

**Affiliations:** 1 Division of Orthodontics, Department of Orofacial Sciences, School of Dentistry, University of California San Francisco, San Francisco, CA 94143, USA; 2 School of Dentistry, University of California San Francisco, San Francisco, CA 94143, USA; 3 Division of Dentistry, Montreal Children’s Hospital and Faculty of Dental Medicine and Oral Health Sciences, McGill University, Montreal, QC H3A 1G1, Canada; 4 Faculty of Dental Medicine and Oral Health Sciences, McGill University, Montreal, QC H3A 1G1, Canada; 5 Craniofacial Tissue Engineering and Stem Cells Laboratory, Faculty of Dental Medicine and Oral Health Science, McGill University, Montreal, QC H3A 1G1, Canada

**Keywords:** biomaterials, bone remodeling, orthodontic-induced root resorption, orthodontic tooth movement

## Abstract

Orthodontic tooth movement (OTM) occurs with the application of a controlled mechanical force and results in coordinated tissue resorption and formation in the surrounding bone and periodontal ligament. The turnover processes of the periodontal and bone tissue are associated with specific signaling factors, such as Receptor Activator of Nuclear factor Kappa-β Ligand (RANKL), osteoprotegerin, runt-related transcription factor 2 (RUNX2), etc., which can be regulated by different biomaterials, promoting or inhibiting bone remodeling during OTM. Different bone substitutes or bone regeneration materials have also been applied to repair alveolar bone defects followed by orthodontic treatment. Those bioengineered bone graft materials also change the local environment that may or may not affect OTM. This article aims to review functional biomaterials that were applied locally to accelerate OTM for a shorter duration of orthodontic treatment or impede OTM for retention purposes, as well as various alveolar bone graft materials which may affect OTM. This review article summarizes various types of biomaterials that can be locally applied to affect the process of OTM, along with their potential mechanisms of action and side effects. The functionalization of biomaterials can improve the solubility or intake of biomolecules, leading to better outcomes in terms of increasing or decreasing the speed of OTM. The ideal timing for initiating OTM is generally considered to be 8 weeks post-grafting. However, more evidence is needed from human studies to fully understand the effects of these biomaterials, including any potential adverse effects.

## 1. Introduction

Orthodontic treatment has a growing demand among the general population due to the increasing social awareness of dental and facial esthetics. However, lengthening the duration of orthodontic treatment, especially in adult patients, can cause various oral health issues, such as root resorption, compromised oral hygiene, white spot lesions, gingivitis, and even periodontitis. Additionally, it can also increase the financial burden on both patients and orthodontists [1]. The duration is affected by the severity of malocclusion, treatment planning, patient compliance, orthodontic treatment mechanics, and the velocity of tooth movements [2]. Among these factors that can affect the treatment duration, one of the best ways to shorten treatment time is by increasing the velocity of orthodontic tooth movement (OTM) to a rate higher than its usual rate of 0.8–1.2 mm/month [3]. However, speeding up OTM can sometimes be undesirable, as it may cause unwanted anchorage loss and relapse post-treatment. In these scenarios, deceleration or inhibition of OTM, on the contrary, is preferable.

Several biomaterials or biosubstances, such as hormones, ligands, and growth factors, involved in the physiological processes of OTM were applied locally as stimuli to the paradental tissues to change the pace of OTM [4,5,6,7,8,9,10,11,12,13,14,15,16,17,18,19,20,21,22]. Some naturally extracted herbal medicines [23,24,25,26,27] or synthesized biomaterials, such as gelatin-reduced graphene oxide [28] that affects bone remodeling, were also investigated for the application of accelerated or decelerated OTM with different extents of root resorption. As one of the potential risks of OTM, root resorption can occur simultaneously when the process of OTM is intervened, especially in accelerated OTM. Different dosages or functionalization of the biomaterials were also tested in some studies to minimize root resorption while controlling the speed of OTM, which will be discussed and summarized in this review.

**Figure 1:**
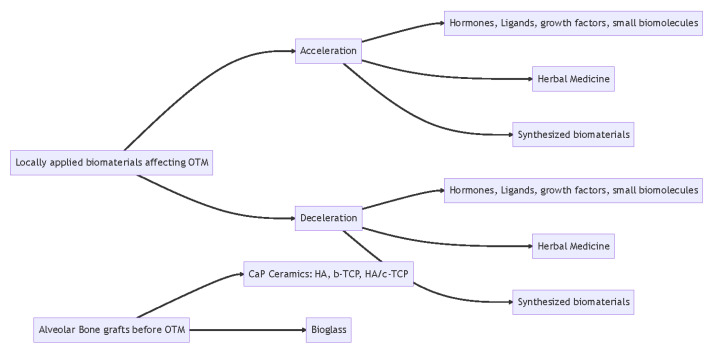
Outlines of the biomaterials affecting OTM.

In addition, OTM is achieved by the remodeling of alveolar bone in response to mechanical loading. Moving teeth into an edentulous area of the dental arch with insufficient bone volume can be challenging and problematic, especially in patients with an alveolar cleft, post-extraction ridge resorption, and periodontal diseases [29]. Therefore, bone grafting for ridge augmentation may be needed before OTM. Alveolar bone grafting materials generally fall into three categories: autografts (e.g., iliac crest, cranium, tibia, rib, and mandibular symphysis), allografts or xenografts, and synthetic bone substitutes (e.g., bioceramics, polymers, or biocomposites) [30]. As autografts mostly show good results of OTM and allografts or xenografts have the potential risks of causing immunologic reactions, this article mainly focuses on the discussion of bone substitutes and their effects on OTM (Figure 1).

The majority of review articles on OTM focus on strategies to increase or decrease the speed of OTM, rather than exploring the use of biomaterials. However, there has been a rapid expansion in the use of biomaterials in orthodontics, some of which show promise for use alone or in combination with bioactive molecules to improve clinical outcomes and better control OTM. It is important to review these biomaterials and their potential side effects, as this can offer valuable insights into the development and enhancement of functional biomaterials for regulating OTM.

## 2. Mechanism of Orthodontic Tooth Movement

Mechanical loads to teeth cause responses of both mineralized and non-mineralized tissues [31]. The response of mineralized tissue often refers to alveolar bone remodeling that causes bone resorption on the compression side and bone deposition on the tension side [1]. Bone resorption is mainly mediated by osteoclasts, whereas bone formation is mainly mediated by osteoblasts [32]. The elucidation of several biochemical processes involved in osteoclastogenesis or osteoblastogenesis offers the ability to target stages of the pathways for the regulation of OTM. During OTM, osteocytes are generally considered first activated by a local strain at the cell membrane in response to mechanical forces [33]. According to Verborgt el al. [34] and Bonewald [35], osteocytes may also send signals for activation of the bone resorption cascade through the expression of activator for Receptor Activator of Nuclear factor Kappa-β Ligand (RANKL) as one of the key factors in bone remodeling. Several signal molecules or biomaterials targeting the RANK/RANKL/OPG pathway, such as TNF-α, PGE2, EGF, etc., were studied for their effects on OTM [4,5,6,13].

**Figure 2:**
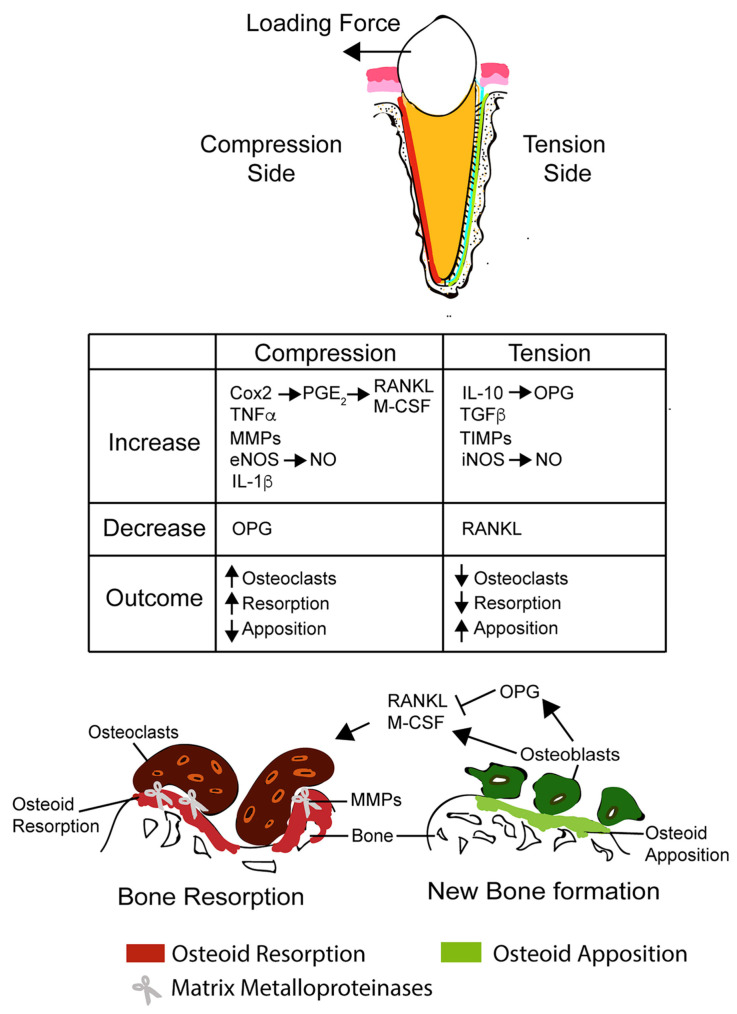
Signal Pathway associated with OTM. The figures are ©2018, Li Y., Jacox L., et al. (https://doi.org/10.1016/j.kjms.2018.01.007) used under an Attribution-NonCommercial-NoDerivatives 4.0 International (CC BY-NC-ND 4.0) License: https://creativecommons.org/licenses/by-nc-nd/4.0/ [40].

In addition to bone tissue, the extracellular matrix (ECM) also reacts to external mechanical loads and undergoes remodeling [36,37]. Several enzymes, such as serine proteases, aspartate proteases, cysteine proteases, and matrix metalloproteinases, are involved in the degradation of collagen and other macromolecules in the ECM as part of the force-induced periodontal ligament (PDL) remodeling [38]. Biomaterials, such as ethylene-vinyl-acetate 40, incorporated with bioactive molecules that affect the activities of these enzymes can also potentially change the biochemical process of OTM [17]. Other non-mineralized responses during OTM include the re-organization and neovascularization of blood vessels and nerve fibers in PDL. Promoting these non-mineralized responses can also enhance or reduce osteogenesis or periodontal ligament (PDL) remodeling, which can lead to changes in the paradental response to OTM [39] (Figure 2).

## 3. Accelerating Orthodontic Tooth Movement

On average, orthodontic treatment lasts around twenty-four months [2]. As mentioned, increased duration of orthodontic treatment is associated with undesired side effects, such as root resorption and increased risk of caries [1]. Therefore, accelerating orthodontic treatment is beneficial for patients, not only in reducing their financial burden with shorter treatment time but also in minimizing the side effects associated with orthodontic treatment. Various methods, such as low-level laser therapy, corticotomy, electrical current, pulsed electromagnetic fields, and dentoalveolar or periodontal distraction surgeries, have been studied for their efficacy in hastening OTM [41]. Long, H. et al. have reviewed and concluded that among all the interventions, a corticotomy is effective and safe for increasing the speed of OTM [41]. In contrast, the other interventions were ineffective or promising due to lack of evidence [41]. However, a corticotomy is an invasive procedure, which raises interest in finding biomaterials that can be applied locally with less invasive administration methods. Among these biomaterials, several hormones, ligands, growth factors, herbal medicines, and even some synthetic materials have been studied to determine their efficacy in increasing OTM while minimizing undesired side effects.

### 3.1. Hormones, Ligands, Growth Factors, and Biomaterials

#### 3.1.1. Prostaglandin E2

Prostaglandins are hormone-like substances produced by the body and involved in numerous biochemical processes. Specifically, prostaglandin E2 (PGE2) is a potent regulator of bone metabolism and exogenous PGE2 has been found to increase transcription and translation of RANKL and hence increase osteoclastogenesis. Different studies using different dosages of PGE2 injected in rats reported statistically significant increased rates of OTM; however, the side effect of root resorption was observed [4,5,6]. Two studies by the same group investigated the use of calcium gluconate or thyroid hormone in conjunction with PGE2 to determine if the undesired side effects of root resorption could be minimized [7,8]. The studies showed that when delivered together, thyroid hormone and PGE2 demonstrated a synergistic effect that accelerated OTM while also decreasing root resorption [8]. A systematic review found that injecting 100 µg PGE2 resulted in a mild-to-moderate increase in OTM, whereas a more consistent and moderate-to-high increase in OTM was observed with the combination therapy of calcium and thyroxine biomaterials [9]. Additionally, this combination therapy led to a simultaneous decrease in the magnitude of root resorption [9]. Different study designs and OTM models used by various studies are summarized in Table 1 and found to be similar, making the effects of the injected solutions easily comparable to determine the best application method in vivo [4,5,6,7,8,10,11,12,13].

#### 3.1.2. Epidermal Growth Factor

Epidermal growth factor (EGF) is an endogenous polypeptide growth factor involved in bone metabolism and has been shown to be involved in tooth eruption. It is expressed in the dental follicle and alveolar bone during the pre-eruption phase of a tooth suggesting its involvement in osteoclast functions [42]. The exact mechanism of its role during OTM has not been established yet. However, studies have found that EGF can be employed to increase OTM. In both experimental designs, EGF was injected submucosally adjacent to the tooth on which the orthodontic force was exerted [10,12]. Delivered in liposomes, EGF was found to increase bone resorption and hence tooth movement [10]. Another study, also delivering EGF in liposomes, found an increase in RANKL expression through the detection of osteoclasts using Tartrate-resistant acid phosphatase (TRAP) staining [12]. The use of EGF alone yielded inconsistent results. The hydrophobic nature of the liposomes facilitated the transport of the EGF through the cell membrane of osteoclasts, osteoblasts, and bone marrow stromal cells and increased the molecule’s bioavailability by slowing down its metabolism, resulting in increased potency of EGF in enhancing OTM [9,10].

#### 3.1.3. Fibroblast Growth Factor

Basic fibroblast growth factor (bFGF) is a protein involved in angiogenesis, tissue remodeling, and the function of osteoblast and osteoclast that has been investigated and found effective in hastening OTM [13]. The positive effects of bFGF were found to be dose-depending with a submucosal injection of 1000 ng to be the most effective dosage in rats. The injection was done in the buccal vestibule close to the mesial root of the first molar. bFGF is thought to enhance OTM by decreasing the lag phase during which blood flow is decreased following the application of orthodontic forces to the PDL through its stimulation of angiogenesis, osteoclasts and osteoblasts [13].

#### 3.1.4. RANKL

RANKL is a protein that binds the RANK receptor on osteoclasts and exhibits an active role in increased bone turnover. It is often implicated in bone resorption occurring on the pressure side during OTM and has been observed to increase the gingival crevicular fluid in humans of people undergoing orthodontic treatment. However, delivery of exogenous RANKL can also have undesired side effects, such as root resorption or even systemic loss of skeletal bones. Studies have therefore focused on elucidating methods to deliver RANKL in a controlled and sustained fashion to limit the side effects while increasing OTM. One study determined that the RANKL NF-hydrogel was capable of slowly delivering bioactive RANKL protein for up to 48 h in vitro [43]. This allows future studies to investigate the use of NF-hydrogels to release RANKL in vivo. A more recent study showed a linear release of RANKL using RANKL adsorbed on poly(lactic acid-co-glycolic acid) microspheres embedded in an aqueous hydroxyethyl cellulose carrier gel in vivo [11]. This study reported an increase in OTM with no record of significant root resorption [11].

#### 3.1.5. Other Hormones and Growth Factors

Other hormones and growth factors, such as osteocalcin, Vitamin D, parathyroid hormone, thyroid hormone, vascular endothelial growth factor (VEGF), platelet-rich concentrates, and recombinant human transforming growth factor-β1, have also been investigated for their effects on OTM but have shown no significant results or low-quality evidence [9].

### 3.2. Herbal Medicine Biomaterials

#### Asperosaponin VI

Asperosaponin VI (ASA VI) is the active ingredient in Dipsacus asper Wall extract, a Chinese herbal medicine traditionally used in the treatment of rheumatic arthritis, lower back pain, and bone fractures [23]. It has been determined to ameliorate bone histomorphology by increasing bone density and the number of bone trabeculae [23,44]. The orthodontic model consisted of a NiTi coil connecting the bilateral maxillary molars to the central incisors that were linked together using a metal band to increase anchorage [23]. The force delivered was 40 g [23]. Experiments where 10 mg/lg of ASA VI was injected locally, submucoperiosteally, and buccal to the right and left maxillary first molars in rats showed a significant increase in OTM in comparison with the control group [23]. ASA VI increases bone resorption on the pressure side shown by an increased expression of RANKL while also aiding in bone deposition on the tension side, shown by an increase in bone density and trabecular spacing [23].

### 3.3. Synthetic Biomaterials

#### Graphene Oxide

Jiao et al. synthesized a biocompatible gelatin-reduced graphene oxide (GOG) to increase the solubility of graphene oxide [28,45]. The group utilized gelatin for this chemical reaction due to its biocompatibility, non-toxicity, and being environmentally friendly without compromising its drug delivery function and to determine its effect on osteogenesis [45]. This functionalized biomaterial showed good biocompatibility in vitro. It was also shown that GOG could induce a local hypoxic microenvironment at the right dosage and hence induces angiogenic differentiation of the mesenchymal stem cells, in turn promoting bone and tissue repair [28]. When injected with GOG submucosally, they exhibited increased OTM through increased osteoclastogenesis, osteoblastogenesis and angiogenesis followed by rapid osteogenesis compared to rats injected with PBS [28].

## 4. Decelerating Orthodontic Tooth Movement

Desirable OTM usually includes precise control of each tooth, minimizing the movement of certain teeth while maximizing the movement of others. In addition, as the final phase of orthodontic treatment, preventing relapse has been a challenge for post-orthodontic retention. Various approaches have been utilized to prevent relapse or provide anchorage, including fixed or removable retainers, periodontal surgery, and local injection of drugs. Moreover, several biomaterials, such as polymers, microparticles, or exosomes, can be applied as a potential delivery vehicle to achieve a prolonged release and therefore provide a better clinical outcome for OTM inhibition [14,15,16,17].

### 4.1. Hormones, Ligands, and Growth Factors

#### 4.1.1. Adiponectin

Adiponectin is a hormone and adipokine protein which is involved in a variety of physiological processes, including metabolic and immune responses [46]. Adiponectin receptors are found to be expressed in mouse gingival fibroblasts, human gingival fibroblasts, and human PDL cells, in addition to osteoblasts and osteoclasts [47,48]. Adiponectin may influence bone metabolism via several mechanisms in bone cells. Adiponectin was shown to stimulate the proliferation and differentiation of osteoblasts [49,50], in addition to favoring the differentiation of mesenchymal stem cells towards the osteoblastic lineage [51]. Several previous studies have also demonstrated a suppressive effect of adiponectin on osteoclasts [41,52,53]. A study by Haugen et al. showed that repetitive submucosal administration of adiponectin resulted in a reduction of tooth movement in rats [54]. The effect was shown to be dosage-dependent and local. More importantly, there were no detectable changes in bone density, periodontal cell number or collagen count after the local injection of adiponectin.

#### 4.1.2. Osteoprotegerin

Osteoprotegerin (OPG) acts as the decoy receptor and competes with RANK for RANKL binding, resulting in the inhibition of terminal stages of osteoclasts and induction of apoptosis. The multifunctional roles of RANK, OPG and RANKL may provide an important link between bone remodeling, OTM, and root resorption [55]. Several studies have shown that the injection of recombinant OPG protein significantly enhances posterior orthodontic anchorage during OTM and diminishes relapse after OTM in rats [18,19,20]. Sydorak et al. took advantage of emerging biomaterial technologies and encapsulated OPG in polymer/PLGA microspheres using a double emulsion technique [15]. With controlled microsphere release kinetics, a single injection of microsphere-encapsulated OPG significantly enhanced orthodontic anchorage, while the single injection of non-encapsulated OPG did not. This is a great example of leveraging bioengineered biomaterials as a controlled-release vehicle. More importantly, the anchorage effect remained to be local where the injection of encapsulated OPG inhibited molar mesialization but did not inhibit the movement of the incisor. Nor did the encapsulated OPG alter alveolar or femur bone volume fraction, density, or mineral content.

#### 4.1.3. Nitric Oxide

Nitric oxide (NO) is a water-soluble, gaseous, short-lived signaling molecule that plays multifaceted roles in a broad range of physiological and pathological processes in mammals [56]. NO also plays an essential role in bone homeostasis regulation, which was previously hypothesized to mediate OTM [57]. Releasing from endothelial cells, NO leads to the production of cyclic guanosine monophosphate (cGMP) which has paracrine effects on nearby smooth muscle cells resulting in a vasodilation response. In addition to its vasodilatory effect, NO is known to alter inflammatory cytokine and chemokine production, alter osteoblast and osteoclast bone anabolism/catabolism, and enhance vascularization and blood flow. Different studies have investigated and provided insight into the regulatory role of NO in OTM [14,21]. A literature review concluded that the administration of the NO precursor and NO synthase (NOS) inhibitor increased and reduced tooth movement in animal models, respectively [21]. Crawford et al. from another study utilized a locally injected NO-releasing silica nanoparticles to establish prolonged NO effects on tooth movement [14]. Crawford concluded that NO released from S-nitrosothiol-containing nanoparticles inhibits the movement of teeth adjacent to the site of the nanoparticle injection for 1 week [14]. Regardless of the promising outcome, further research is required to elucidate the underlying mechanisms and clinical application of NO in OTM.

#### 4.1.4. Matrix Metalloproteinase Inhibitor: Ilomastat

During bone remodeling, components of the extracellular matrix, such as collagen, are degraded and removed, and new components are synthesized and reposited. Matrix metalloproteinases (MMPs) are a family of zinc-dependent endopeptidases comprising over 25 enzymes regulating many biological processes, including the regulation of bone remodeling. Recent studies indicate that the activity of MMPs is important for the activation of osteoclasts in bone resorption. Holliday et al. showed that OTM was effectively inhibited by the local delivery of Ilomastat, a general matrix metalloproteinase inhibitor, with the use of ethylene-vinyl-acetate (ELVAX) 40, a non-biodegradable, non-inflammatory sustained-release polymer [17].

#### 4.1.5. Hydroxyapatite-Incorporated Advanced Platelet-Rich Fibrin

Carbonated hydroxyapatite (CHA) is an ideal biomaterial for bone remodeling enhancement because it has an interconnected porous structure with an inorganic component mimicking bone tissue. It can undergo bone-like remodeling and exhibit excellent osteoconductivity [58]. Platelet-rich fibrin (PRF) is a new generation platelet concentration; it contains high growth factor levels, including transforming growth factors (TGF), vascular endothelial growth factors (VEGF), platelet-derived-growth factors (PDGF), epidermal growth factors (EGF), and insulin like-growth factors (IGF) that play a central role in the bone healing process [59]. Alhasyimi et al. showed that intrasulcular injection of hydrogel CHA incorporated PRF could locally reduce orthodontic relapse in rabbits [22]. Histological analysis revealed that the number of osteoblasts was significantly higher, and that the osteoclast activity was significantly lower in the CHA-aPRF group compared to the other groups.

### 4.2. Small Molecules or Herbal Medicine

#### 4.2.1. Triptolide

Triptolide, a diterpenoid trioxide isolated from the ancient Chinese herb Tripterygium wilfordii Hook F (TWHF), has been widely used to treat autoimmune and inflammatory diseases in China [60,61]. Researchers have found triptolide could inhibit bone loss and upregulate the bone mineral density [62]. Experiments where triptolide was injected locally in rats demonstrated that the amount of tooth movement and the ratio of root resorption were significantly decreased in comparison with the control group [24]. The number of TRAP-positive cells was significantly lower in triptolide-treated groups. Moreover, the expression of RANKL was reduced. In contrast, the expression of OPG was significantly upregulated. On the tension side, the expressions of runt-related transcription factor 2 (RUNX2) and osteocalcin (OCN) were significantly enhanced by triptolide injection. Yang et al. suggested that triptolide may exert a positive effect on osteoblastogenesis and an inhibitory effect on osteoclastogenesis [24].

#### 4.2.2. Resveratrol

Resveratrol (RSV) is a naturally occurring phytoalexin found in grapes, blueberries, peanuts, and other food products derived therefrom (e.g., wine) [63]. This compound has received worldwide attention and has been regarded as a potential drug candidate for a wide range of chronic diseases, such as cardiovascular diseases, autoimmune diseases, and cancer [63,64,65]. Some evidence also indicated that RSV could affect bone metabolism by reducing the formation of osteoclasts while promoting osteoblasts’ formation [66]. A recent study by Liu et al. demonstrated that RSV groups showed a significant decrease in OTM and the orthodontic-induced root resorption (OIRR) ratio [25]. The number of TRAP-positive osteoclasts and the expression of RANKL in periodontal tissue of the RSV groups were significantly inhibited, while the expression of OPG, RUNX2, and OCN was remarkably upregulated. This was considered partly due to RSV’s inhibitory effect on osteoclasts and promoting effect on osteoblasts [25].

#### 4.2.3. Sinomenine

Sinomenine (SIN), a natural alkaloid, is the main component of the traditional Chinese medicine sinomenium acutum. SIN is known for its potent therapeutic effects, such as anti-inflammatory, immunosuppressive, antioxidant, antiarrhythmic and anti-cancer [26,67,68]. Some studies have found that SIN affects bone metabolism. Specifically, it has been reported that SIN can inhibit the differentiation of osteoclasts via RANKL signaling pathways and promote the differentiation of osteoblasts by regulating the Akt/RUNX2 signaling pathway [67,69]. Li et al. tested SIN and its potential effect on OTM [26]. As the rat OTM model has shown, SIN reduced tooth movement as well as root resorption and exerted a positive effect on bone formation in rats. Moreover, SIN promotes the osteogenesis of periodontal ligament stem cells (PDLSCs).

#### 4.2.4. Exosomes-Encapsulated Simvastatin

Recently, there has been a growing interest in simvastatin and its effect on bone. It has previously been reported that the local administration of simvastatin can prevent relapse after OTM in rats and rabbits [27]. However, simvastatin has poor bioavailability, primarily due to its low water solubility. Several studies have supported exosomes as a potential drug carrier, which may improve the efficacy of fat-soluble drugs, such as simvastatin [70]. Liu et al. tested whether simvastatin encapsulated in exosomes can inhibit relapse after OTM [16]. In Liu’s study, exosomal simvastatin was obtained by co-incubation of simvastatin and the exosomes of PDLSCs. The study showed that exosomal simvastatin could increase the solubility of the drug and subsequently enhance its inhibition of relapse after OTM in the rat model.

There are a good number of studies that investigate the inhibitory effects of natural biomaterials or bioengineered materials on orthodontic tooth movement and associated root resorption. Although most animal studies were conducted with a rat OTM model using a closed coil nickel-titanium spring inserted unilaterally between the maxillary first molar and incisors, there are certain levels of variations in the experiment setup. One study used a rabbit OTM model with the spring placed between two incisors [27]. For studies using a rat OTM model, the amount of orthodontic force being applied (from 25 g to 50 g), the duration of tooth movement (from 10 to 28 days), and/or retention period varies greatly across different studies. In addition, the interventions were given differently with different volumes, concentrations, and conditions. For instance, some injections were administrated as a single dose versus others being administrated daily throughout the entire OTM duration. Therefore, it is hard to qualitatively compare the effectiveness of the inhibitory effects across different studies. The studies of the biomaterials used to inhibit OTM are summarized in Table 2.

## 5. Orthodontic Tooth Movement after Bone Grafting

Extractions are routinely performed to gain space in dental arches to alleviate crowding, one of the most common problems in orthodontics. Post-extraction ridge resorption and gingival ingrowth can cause several challenges to OTM since adequate bone supply is essential to achieve the desired result [30]. Other conditions, such as periodontal disease and cleft palate, are also indications of ridge augmentation before moving teeth into the affected area. Amongst several bone graft materials used to repair bony defects, autogenous bone is the gold standard thanks to its biocompatibility and osteogenetic abilities [30]. However, autografts have their shortcomings, the biggest one being the need for a donor site and the potential morbidity associated with the procedure. Autogenous particulate cancellous bone and marrow allow for good results of OTM in the cleft area with adequate periodontal outcomes [71]. Freeze-dried bone allograft (FDBA) and decalcified freeze-dried bone allograft (DFDBA) are the two most popular allografts for cleft repair. DFDBA is considered superior in the context of OTM due to its greater viable bone formation and lower residual graft particles. Bio-Oss, the most prevalent bovine-derived xenograft, has been used extensively in bone reconstruction. The slow degradation of this material would theoretically hinder the OTM. However, Araujo et al. found no difference between the Bio-Oss and control group in terms of root resorption and the rate of OTM [72]. Nonetheless, there is a lack of evidence to use Bio-Oss alone in ridge augmentation in OTM. Although allografts and xenografts do not require a secondary surgical site to harvest the donor’s bone, they can cause immunologic reactions at the recipient site [71]. These drawbacks lead the researchers to investigate synthetic graft materials to find an optimal substitute mimicking natural bone. The current synthetic materials used in bone augmentation prior to orthodontic treatment are mainly calcium phosphate and bioglass.

### 5.1. Calcium Phosphate (CaP) Ceramics: HA, b-TCP, HA/c-TCP

Bone is composed of organic materials and inorganic minerals. Due to the similarity of calcium phosphate materials to bone minerals and their osteoinductivity, they are common synthetic bone substitutes used today in guided bone regeneration. There are three significant compositions of CaP: hydroxyapatite, beta-tricalcium phosphate, and biphasic CaP (HA/c-TCP) [73].

#### 5.1.1. Hydroxyapatite

Hydroxyapatite (HA) is naturally present in the bone and represents 50% of its calcium and phosphorus storage. Due to its high calcium-to-phosphorus ratio, it has a low degradation rate [71]. Commercially available NanoBone is a form of hydroxyapatite treated to optimize biodegradability to simulate the natural bone. In 2006, Proff et al. reported a successful bone augmentation with NanoBone before OTM without significant complications [29]. An animal experiment conducted on three pigs observed root resorption when orthodontic treatment was initiated immediately post-surgery [74]. Subsequently, Reichert et al. reported successful bone augmentation with NanoBone and no significant adverse effects on OTM, such as inflammation and root resorption [39,75]. These results were confirmed by the study from Seifi et al. in 2015, who initiated OTM eight-week post-surgically [76]. They evaluated the root absorption in the NanoBone-grafted site and compared it to the control site that was left to heal naturally. Although root resorption was more prominent in the grafted group, the difference between groups was not statistically significant [75].

#### 5.1.2. Beta-Tricalcium Phosphate Ceramic

Beta-tricalcium phosphate ceramic (b-TCP) has a much faster degradation and absorption due to its lower Ca/P ratio, making it an excellent grafting material before OTM. Multiple studies validate the use of b-TCP as an adequate bone substitute without causing any adverse effect on OTM [76,77,78]. To improve osteogenesis and mineralization, osteogenically induced bone marrow stromal cells were combined with b-TCP as a new synthetic bone substitute. In the experiment by Zhang et al., this tissue-engineered bone produced comparable augmentation results as autologous grafts, whereas b-TCP alone had significantly more absorption of granulated CaP tissue [79,80]. A later study determined that the best initiation time for OTM was eight weeks after bMSCs/β-TCP graft placement. The distance of tooth movement, the number of TRAP-positive cells and the optical density of BMP-2 were significantly lower in the 2-week and 4-week groups. There was, however, no difference between the 8-week and 12-week groups. This timing corresponds to the highest level of osteogenesis [81].

#### 5.1.3. Biphasic Calcium Phosphate

Recently, biphasic calcium phosphate (BCP), a composite of HA and b-TCP, is gaining popularity as it takes advantage of both materials to achieve fast bone regeneration while allowing the development of vasculature and osteoblast attachment thanks to the slowly resorbed HA. BoneCeramic is one example of BCP composed of 60% HA and 40% b-TCP. In one study, BoneCeramic demonstrated retardation of OTM compared with Bio-Oss. This slower OTM could be explained by a higher quality of grafted bone and less root resorption post-OTM [80]. Although BCP offers better bone generation, it is still not comparable to autografts and human xenografts [30].

There is very limited literature on using a combination of stem cells and CaP in the context of OTM. Bone marrow stromal cells (bMSCs) have shown the ability to repair cleft defects. In both Zhang and Tanimono’s studies, combining bMSCs and CaP promotes bone formation and allows a more consistent OTM rate than the CaP graft alone [81,82]. More studies with larger sample sizes are necessary to establish the effect of grafting using tissue-engineered stem cells combined with CaP on OTM.

### 5.2. Bioglass

Bioglass (BG) is a silica-based material having a potent physical affinity with host bone. Its porosity allows the ingrowth of vasculature, and its resorption rate is favourable for osteogenesis [71]. When interstitial fluid contacts the bone graft, silicon ions activate to increase the pH and induce the development of a calcium phosphate layer [74]. Attia et al. observed that not only is OTM not affected in the BG-grafted site, but OTM also enhances osteogenic activities [83]. This enhancement was more prominent in the group where OTM was initiated immediately after grafting surgery compared to the delayed OTM group [84]. Different synthetic bone graft materials used for bone defect in OTM are summarized in Table 3. 

## 6. Conclusions

Several types of biomaterials were used solely or functionalized to change the pace of OTM in different animal models, with little evidence from human studies until now. They were mainly applied submucosally, intraligamentously, intraperitoneally, intrasulcularly, or intraosseously through osteoperforation for local application. The influential effect is dosage-dependent in some materials, such as bFGF and adiponectin. Side effects, such as root resorption, may happen along with accelerated OTM. However, they may be reduced or controlled by incorporating other materials in the system (e.g., Ca in PGE2). At the same time, it can be decreased by applying certain biomaterials inhibiting OTM, such as resveratrol and sinomenine. Functionalization of biomaterials can sometimes increase the solubility or intake of the molecules locally and therefore is preferable for a better outcome.

Multiple bone substitutes were also used for alveolar bone graft and tested for OTM with different extents of root resorption. Most of them do not have adverse effect on OTM, with the controversy of having equal or slower OTM compared to autograft. Generally, 8 weeks post-grafting is considered the best timing to initiate OTM.

More research is needed to prevent harmful outcomes from locally delivered biomaterials, as few reports on their adverse effects have been published. Additionally, the scientific basis of proposed methods to alter tooth velocity needs to be broadened and derived from comprehensive perspective studies.

## Figures and Tables

**Table 1:** Information of studies using biomaterials to accelerate OTM.

Author Ref./Year	Sample Size	OTM Model Animal/Force	Biomaterials (Dosage/Route of Administration)	Outcomes
Cağlaroğlu M, 2012 [4]	45	Rabbit/ 20 g reciprocal force on the maxillary incisor	PGE2 (10 μg/mL /Intravenous, submucosal, or intraligamentous)	1. Increase in OTM in rabbits receiving submucosal and intraligamentous PGE2 injection, with increased OTM observed in rabbits receiving the intraligamentous injection in comparison to the submucosal injection 2. Root resorption not found to be a statistically significant risk but may still be a risk.
Cui J, 2018 [5]	40	Wistar rats/40 g mesial force on maxillary first molar	PGE2 (25 μg/kg), akebiasaponin D (5 mg/kg) and (10 mg/kg)	PGE2 injection yielded increased OTM when compared to injection of akebiasaponin D of 5 mg/kg and similar results to injection of akebiasaponin D 10 mg/kg
Leiker B, 1995 [6]	132	Sprague-Dawley rats/60 g force mesial on the maxillary first molar	PGE2 (0.1, 1, 5, and 10 μg/submucosa	1. Increased OTM with injection of PGE2 with no statistical significance between groups. 2. Increased root resorption associated with increased number of injections and higher concentrations
Seifi M, 2003 [7]	24	Wistar rats/60 g force mesial to the maxillary first molar	PGE2 (0.1 mL of 1 mg/mL)/submucosal + 10% Ca (200 mg/kg)/intraperitoneal	1. PGE2 injection showed increased OTM but PGE2 with Ca showed a higher OTM than the control but less than PGE2 alone 2. No significant difference in root resorption between control, PGE2 or PGE2 and Ca.
Seifi M, 2015 [8]	64	Wistar rats/60 g force mesial to the maxillary first molar	Thyroxine (20 μg/kg)/intraperitoneal PGE2 (0.1 mL of 1 mg/mL)/submucosal 10% Calcium gluconate (200 mg/kg/intraperitoneal)	1. Highest OTM observed in thyroxine and PGE2 groups 2. Root resorption observed with statistical difference in PGE2 groups in comparison to others
Saddi KR, 2008 [10]	32	Holtzman rats/mesial force on the maxillary first molar	EGF within liposome (2 ng/μL) Soluble EGF (2 ng/μL) Liposomes only /submucosal	1. Highest OTM observed in rats injected with EGF or EGF in liposomes. 2. Statistically significant higher number of osteoclasts recruited in group injected with EGF in liposomes
Chang JH, 2020 [11]	24	Wistar rats/5–8 g force mesial to maxillary first molar	RANKL (1 μg): microsphere(1 mg) in 3 μL 10% HEC gel/intraosseous through osteoperforation	1. Increased OTM associated with RANKL injection 2. Decreased tissue density observed but no significant root resorption difference between groups observed
Alves JB, 2009 [12]	96	Holtzman rats/20 g force mesial to maxillary first molar	Empty liposomes EGF (20 ng) EGF-Liposome(20 ng) /submucosal	EGF-liposome injection was associated with statistically significant increased OTM and osteoclast numbers and RANKL expression
Seifi M, 2013 [13]	50	Wistar rats/60 g mesial force to maxillary first molar	bFGF (10 ng, 100 ng, and 1000 ng)/ submucosal	Increased OTM associated with bFGF injections and is dose dependent, with 1000 ng bFGF being the most effective

**Table 2:** Information of studies using biomaterials on OTM inhibition.

Author Ref./Year	Sample Size	OTM Model Animal/Force	Biomaterials (Dosage/Route of Administration)	Outcomes
Crawford D, 2022 [14]	32	Sprague Dawley rats/ 50 g reciprocal force between maxillary 1st molar and incisors for 18 days	S-nitrosothiol (2.2 mg/kg of S-nitrosothoil containing nanoparticles, in saline/intraperitoneal injection immediately prior to orthodontic appliance activation)	Nitric oxide (NO) released from S-nitrosothiol containing nanoparticles inhibited movement of teeth for 1 week.
Sydorak I, 2019 [15]	42	Sprague Dawley rats/ 25 g reciprocal force between maxillary 1st molar and incisors for 28 days	OPG (1 mg/kg non-encapsulated or microsphere encapsulated/intraperitoneal injection/single dose injected mesial to maxillary 1st molar one day prior to OTM)	A single injection of microsphere encapsulated OPG significantly enhanced orthodontic anchorage, while a single injection of non-encapsulated OPG did not.
Liu X, 2022 [16]	32	Sprague Dawley rats/ 50 g reciprocal force between maxillary 1st molar and incisors for 14 days	Simvastatin encapsulated in exosome, simvastatin in saline/submucosal and intraligementary injection	Encapsulating simvastatin into the exosomes derived from PDLSCs improved simvastatin solubility and enhanced the inhibition effect of relapse.
Holliday L, 2003 [17]	N/A	Sprague Dawley rats/ 40 g reciprocal force between maxillary 1st molar and incisors for 10 days	Polymer ELVAX40 impregnated with Ilomastat/intraperitoneal injection	In the presence of Ilomastat, tooth movement on day 10 was significantly inhibited. Initial tipping and the lag phase were not significantly different.
Alhasyimi A, 2018 [22]	45	Rabbits/ 50 cN reciprocal force between lower incisors for 14 days	Carbonated hydroxyapatite-incorporated advanced platelet-rich fibrin (CHA-aPRF)/intrasulcular injection every 7 days	Intrasulcular injection of hydrogel CHA incorporated aPRF locally reduced the orthodontic relapse in rabbits.
Yang F, 2019 [24]	48	Wistar rats/ 50 g reciprocal force between maxillary 1st molar and incisors for 14 days	Triptolide (30 or 15 µg/kg/day/ intraperitoneal injection)	The amount of tooth movement and the ratio of root resorption area were significantly decreased in the triptolide-treated rats.
Liu X, 2020 [25]	36	Wistar rats/ 50 g reciprocal force between maxillary 1st molar and incisors for 14 days	Resveratrol (10 or 5 mg/kg/day, dissolved in CMC/gavage)	The RSV groups showed a significant decrease in the distance of OTM and orthodontic induced root resorption ratio.
Li H, 2022 [26]	54	Wistar rats/ 50 g reciprocal force between maxillary 1st molar and incisors for 14 days	Sinomenine (40 or 20 mg/kg/day/intraperitoneal injection)	The tooth movement and root resorption of sinomenine groups were reduced.
Haugen S, 2017 [54]	24	Wistar rats/ 0.5 N reciprocal force between maxillary 1st molar and incisors for 14 days	Adiponectin (2 or 0.2 µg every third day for 14 days/intraperitoneal injection)	Submucosal injections of adiponectin prevented experimental tooth movement in rats. The effect was dosage-dependent and local.

**Table 3:** Different Synthetic Bone Graft Materials for Bone Defects Augmentation in OTM.

Author Ref./Year	Sample Size	OTM Model Animal/Force	Grafting Materials	Outcomes
Möhlhenrich SC, 2021 [30]	21	Wistar Rat/NiTi coil springs (0.14 N)	β-TCP/HA (beta-tricalcium phosphate/hydroxyapatite) vs. Autograft & Human Xenograft	1. Autograft has the best bone integration and the β-TCP/HA the least 2. OTM has a secondary role in the remodeling process of grafted bone
Araujo MG, 2001 [72]	5	Beagle Dog/closed coil spring of 30 to 50 cN	Bio-Oss	1. OTM occurred in grafted site without complication 2. Zone of the ridge with OTM shows degradation of Bio-Oss 3. Zone without OTM inactive filler material remains after 12 months
Gedrange T, 2010 [74]	1	Pig (Sus scrofa domesticus)/ 1–2 N on each adjacent tooth to the right mandibular premolar	HA (Nano-Bone^®^ of Artoss GmbH, Germany)	1. OTM through grafted sites could increase the risk of root resorption 2. Different resorption changes at apical, medial, and coronal root sections
Reichert C, 2011 [75]	3	Human/ NiTi closed coil springs (200 g)	HA (Nano-Bone^®^ of Artoss GmbH, Germany)	1. Gingival invagination causes delayed OTM in control sites 2. OTM was possible and faster in grafted area without adverse effect (e.g., Root resorption or inflammation)
Seifi M, 2015 [76]	4	Beagle Dog/NiTi closed coil springs (159 g)	HA (NanoBone^®^ non-sintered porous nano-crystalline hydroxyapatite)	1. No difference between grafted site compared to non-grafted site on OTM 2. The use of synthetic bone substitute can induce neovascularization and osteogenesis 3. Grafting with HA does not have a major impact on the amount of root resorption post-OTM
Hossain MZ, 1989 [77]	20	Beagle Dog/Coil spring adjusted to achieve OTM of of 2 mm per month	β-TCP (beta-tricalcium phosphate ceramic; Synthograft^®^) vs. autogenous PMCB (particulate marrow and cancellous bone)	1. No significant difference compared to PMCB 2. No adverse effect on OTM
Sheats RD, 1991 [78]	12	Cat/NiTi close coil spring (100 g)	β-TCP (beta-tricalcium phosphate ceramic; Synthograft^®^)	1. No difference between grafted site compared to non-grafted site on OTM
Hossain MZ, 1996 [79]	9	Beagle Dog/Open coil spring activated to achieve 2 mm OTM per month	β-TCP vs. autogenous PMCB (particulate marrow and cancellous bone)	1. TCPC exhibits more biodegradability and remodeling ability, resulting in less root resorption compared to PMCB
Zhang FF, 2019 [80]	40	New Zealand Rabbit/NiTi closed coil springs (80 g)	bMSCs/β-TCP	1. 8 weeks post -surgery is the best initiation time for OTM
Zhang D, 2011 [81]	6	Beagle Dog/NiTi closed coil springs (50 g)	bMSCs/β-TCP vs. β-TCP vs. Autograft	1. Synthetic bone grafts have no effect on OTM bMSCs/β-TCP promotes new bone formation and mineralization compared to β-TCP alone
Ru N, 2016 [82]	40	Sprague Dawley Rat/NiTi coil springs (10 g)	β-TCP/HA (BoneCeramic Straumann, Basel, Switzerland) vs. natural bovine cancellous bone particles (Bio-Oss; Geistlich Pharma, Wolhusen, Switzerland)	1. BoneCeramic produces the slowest OTM 2. BonCeramic causes the least root resorption compared to Bio-Oss
Attia MS, 2012 [83]	15	Human/10–15 g per tooth	Bio-Glass	1. Combined orthodontic/regenerative therapy promotes periodontal regeneration
Tanimoto K, 2015 [84]	3	Beagle Dog/Elastic chain (100 g)	MSC+ CAP (bone marrow-derived mesenchymal stem cells + carbonated hydroxyapatite) vs. CAP	1. MSC+ CAP allows for OTM at a constant rate compared to the CAP grafted site that resulted in OTM at various rate
Möhlhenrich SC, 2022 [85]	21	Wistar Rat/NiTi coil springs (0.14 N)	β-TCP/HA (beta-tricalcium phosphate/hydroxyapatite) vs. Autograft & Human Xenograft	1. All three graft materials have a similar effect on OTM and root resorption
Machibya SC, 2018 [86]	24	Beagle Dog/NiTi closed coil springs (150 g)	β-TCP vs. Bio-Oss	1. Initiation after 1 month: Bio-Oss has better radiologic features but slower OTM 2. Initiation after 2 months: β-TCP has better radiographic features and faster OTM compared to early OTM in the β-TCP group.
Jiang S, 2020 [87]	9	Beagle Dog/NiTi closed coil springs (150 g)	BioCaP BMP2-functionalized biomimetic calcium phosphate (BioCaP) vs. deproteinized bovine bone (DBB)	1. BioCaP graft promotes alveolar bone healing and reduces root resorption during OTM compared to DBB
Zhang J, 2006 [88]	40	Wistar Rat/NiTi coil springs (0.39 N)	Bio-Glass	1. Good integration of the bone graft material 2. No difference between grafted site compared to non-grafted site on OTM

## Data Availability

No new data were created or analyzed in this study. Data sharing is not applicable to this article.
